# *rac*-(1S*,4aS*,8aS*)-4a-Hy­droxy-2-methyl­perhydro­spiro­[isoquinoline-4,1′-cyclo­hexa­n]-2′-one

**DOI:** 10.1107/S1600536813007010

**Published:** 2013-04-05

**Authors:** Sorho Siaka, Anatoly T. Soldatenkov, Anastasia V. Malkova, Svetlana A. Soldatova, Victor N. Khrustalev

**Affiliations:** a Institut National Polytechnique Félix Houphouët-Boigny, Enseignant-Chercheur à l’INP-HB de Yamoussoukro, BP 991 Yamoussoukro, Côte d’Ivoire; b Organic Chemistry Department, Russian Peoples Friendship University, Miklukho-Maklaya St. 6, Moscow 117198, Russia; c X-Ray Structural Centre, A.N. Nesmeyanov Institute of Organoelement Compounds, Russian Academy of Sciences, 28 Vavilov Street, B-334, Moscow 119991, Russian Federation

## Abstract

In the title compound, C_15_H_25_NO_2_, all three six-membered rings adopt chair conformations. The cyclo­hexane and piperidine rings within the perhydro­isoquinoline are *trans*–*trans* fused. The N atom has a trigonal–pyramidal geometry (the sum of the bond angles is 328.0°). The methyl substituent occupies the sterically preferrable equatorial position. In the crystal, mol­ecules form infinite [100] chains *via* O—H⋯N hydrogen bonds.

## Related literature

For general background to the synthesis, chemical properties and applications in medicine of the title compound, see: Plati & Wenner (1949 ▶); Ellefson *et al.* (1978 ▶); Soldatenkov *et al.* (2009 ▶). For related compounds, see: Plati & Wenner (1950 ▶); Soldatenkov *et al.* (2008 ▶); Soldatova *et al.* (2010 ▶); Siaka *et al.* (2012 ▶).
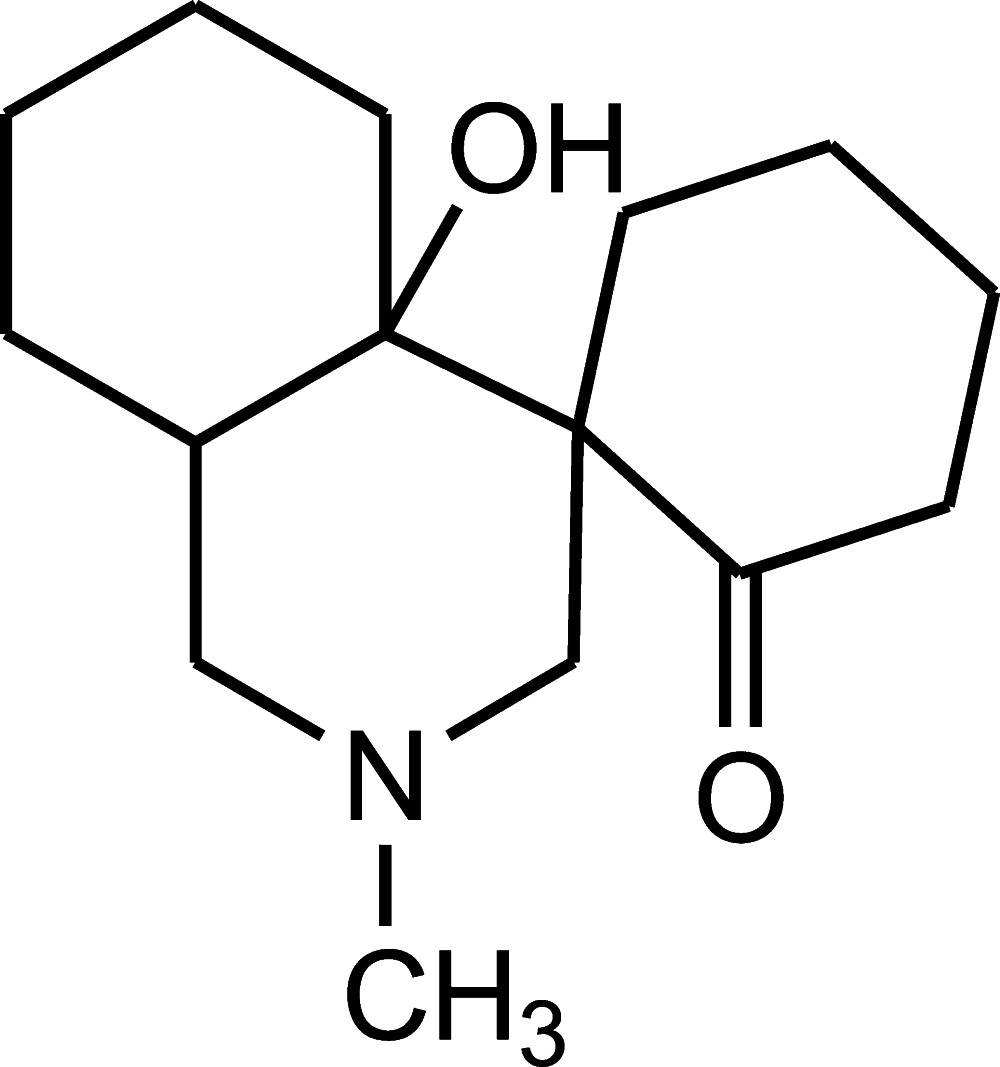


## Experimental

### 

#### Crystal data

C_15_H_25_NO_2_
*M* *_r_* = 251.36
Monoclinic, 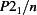

*a* = 5.8438 (2) Å
*b* = 18.5756 (7) Å
*c* = 12.2148 (5) Å
β = 95.116 (1)°
*V* = 1320.66 (9) Å^3^
*Z* = 4
Mo *K*α radiation
μ = 0.08 mm^−1^
*T* = 100 K
0.30 × 0.30 × 0.20 mm

#### Data collection

Bruker APEXII CCD diffractometer
Absorption correction: multi-scan (*SADABS*; Sheldrick, 2003 ▶) *T*_min_ = 0.976, *T*_max_ = 0.984
17142 measured reflections
3852 independent reflections
3221 reflections with *I* > 2σ(*I*)
*R*_int_ = 0.028

#### Refinement

*R*[*F* ^2^ > 2σ(*F* ^2^)] = 0.037
*wR*(*F* ^2^) = 0.102
*S* = 1.00
3852 reflections
167 parameters
H atoms treated by a mixture of independent and constrained refinement
Δρ_max_ = 0.48 e Å^−3^
Δρ_min_ = −0.18 e Å^−3^

### 

Data collection: *APEX2* (Bruker, 2005 ▶); cell refinement: *SAINT* (Bruker, 2001 ▶); data reduction: *SAINT*; program(s) used to solve structure: *SHELXTL* (Sheldrick, 2008 ▶); program(s) used to refine structure: *SHELXTL*; molecular graphics: *SHELXTL*; software used to prepare material for publication: *SHELXTL*.

## Supplementary Material

Crystal structure: contains datablock(s) global, I. DOI: 10.1107/S1600536813007010/rk2397sup1.cif

Structure factors: contains datablock(s) I. DOI: 10.1107/S1600536813007010/rk2397Isup2.hkl

Supplementary material file. DOI: 10.1107/S1600536813007010/rk2397Isup3.cml

Additional supplementary materials:  crystallographic information; 3D view; checkCIF report

## Figures and Tables

**Table 1:** Hydrogen-bond geometry (Å, °)

*D*—H⋯*A*	*D*—H	H⋯*A*	*D*⋯*A*	*D*—H⋯*A*
O1—H1⋯N3^i^	0.830 (12)	2.195 (12)	2.8967 (10)	142.3 (12)

Symmetry code: (i) 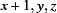.

**Fig. 1.**
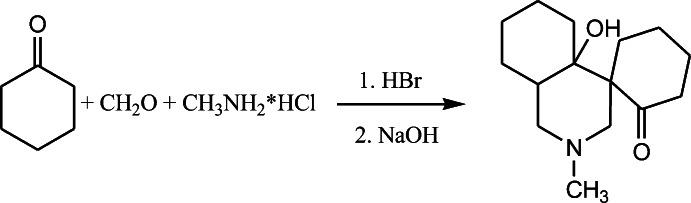
The cascade condensation of cyclohexanone with formaldehyde and methylamine.

**Fig. 2.**
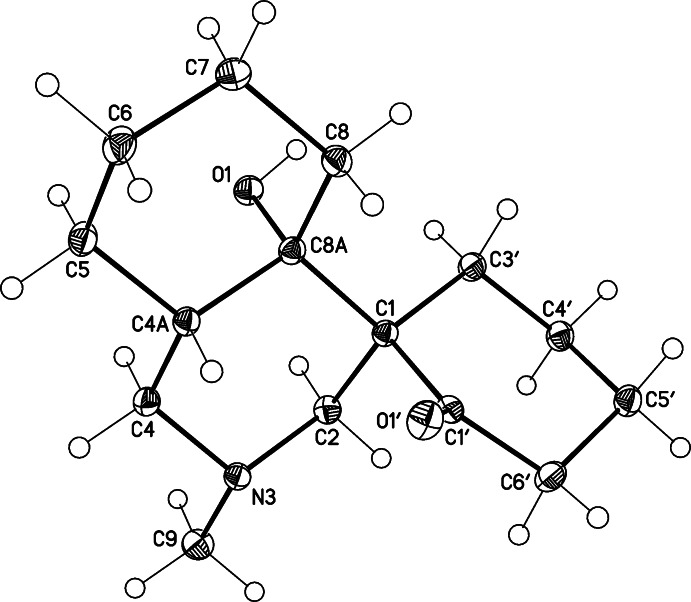
Molecular structure of I with the atom numbering scheme. Displacement ellipsoids are shown at the 50% probability level. H atoms are presented as small spheres of arbitrary radius.

**Fig. 3.**
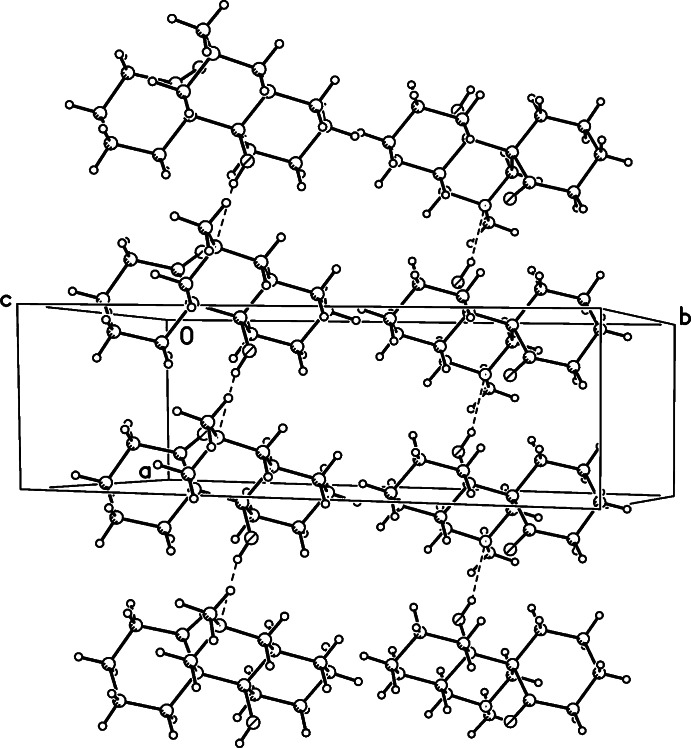
A portion of the crystal structure showing the packing of the H–bonded infinite chains of I. The intermolecular O—H···N hydrogen bonds are depicted by dashed lines.

## supplementary crystallographic information

### Comment

It is well known that 3–aroyl–4–arylpiperidin–4–ols are intermediate products in the synthesis of important antihistaminic agents (Plati & Wenner, 1950; Ellefson *et al.*, 1978). Such piperidols can be prepared in two steps *via* the condensation of the corresponding acetophenones with formaldehyde and alkylamines by heating of these mixtures in 36% HCl solution and the subsequent intramolecular cyclization of the yielded double Mannich bases under action of bases (Plati & Wenner, 1949).

However, we found that use of α–tetralone - the cyclic analogue of acetophenone (Soldatenkov *et al.*, 2008) in the analogous syntheses results in very complex multicomponent mixtures instead of the expected γ–piperidol derivatives. These mixtures contain only trace quantities of the expected piperidols (identified by *LC*–*MS* method). It is interesing to note that heating of the analogous double Mannich base prepared from α–tetralone (Soldatenkov *et al.*, 2009; Soldatova *et al.*, 2010; Siaka *et al.*, 2012) in HBr solution gives the expectable product of the cyclization, but in the dehydrated form. Its structure comprising the spiro–fused hexahydrobenzo[*f*]isoquinoline and tetrahydronaphthalenone systems was unambiguously established by X–ray diffraction study (Siaka *et al.*, 2012). Thus, such a molecule appears to be much more stable than its 10*b*–hydroxy predecessor. Hence, we were not sure that structure of the main product would be chemically preferable in the analogous multicomponent condensation if we would use cyclohexanone as the ketone component (heating in HBr solution; Fig. 1). We have found that, in this case, 8*a*–hydroxy–3–methylperhydrospiro[isoquinoline–1,2'–cyclohexan]–1'–one is formed, which is very stable towards dehydration in acidic media. The first step of this cascade process is the formation of the double Mannich base, and the second one is aldol–type intramolecular cycloaddition of the two cyclohexenone moieties to each other. The structure of the spiro–derivative, C_15_H_25_NO_2_, (I) was unambiguously established by X–ray diffraction study.

The molecule of I comprises spiro–fused perhydroisoquinoline and cyclohexanone systems (Fig. 2). All the three saturated six–membered rings adopt chair conformations. The cyclohexane and piperidine rings within the perhydroisoquinoline are fused in *trans*–*trans* type. The nitrogen atom has a trigonal–pyramidal geometry (sum of the bond angles is 328.0 (2)°). The methyl substituent occupies the sterically preferrable equatorial position.

The molecule of I possesses three asymmetric centers at the C1, C4A and C8A carbon atoms and can have potentially eight diastereomers. The crystal of I is racemic and consists of enantiomeric pairs with the following relative configuration of the centers: *rac*–1*S**,4*aS**,8*aS**.

In the crystal, molecules form infinite [1 0 0] chains *via* the intermolecular O1—H1···N3^i^ hydrogen bonds (Fig. 3, Table 1). The chains are arranged at van–der–Waals distances. Symmetry code: (i) *x*+1, *y*, *z*.

### Experimental

A mixture of methylamine hydrochloride (14.0 g, 0.2 mol), cyclohexanone (41.4 ml, 0.4 mol) and formaldehyde (31 ml of 40% solution in water) in 48% HBr (80 ml) was boiled for 6 h. The reaction mixture was then cooled, poured into cold water (200 ml) and stirred at 293 K for 7 h. The pH of the mixture was then brought at 9, and the expected product was extracted by ether. The obtained extract was washed with water (50 ml), dried over disodium sulfate. After the solvent evaporation, the residue was purified by re–crystallization from ethanol to give 16.75 g of colorless crystals of I. Yield is 28%. M.p. = 437–439 K. IR (KBr), ν/cm^-1^: 3452, 3400, 1698. ^1^H NMR (CDCl_3_, 400 MHz, 300 K): δ = 1.19–1.65 (m, 7H, C—CH_2_), 1.68 (t, 2H, ^3^*J* = 11.7, C—CH_2_), 1.88 (t, 1H, ^3^*J* = 12.0, C—CH_2_), 1.94–2.06 (m, 4H, C—CH_2_), 2.18 (s, 3H, CH_3_), 2.22 (m, 1H, C—CH), 2.40 (d, 1H, ^3^*J* = 12.0, N—CH_2_), 2.43 (m, 1H, NCH_2_), 2.55–2.70 (m, 3H, NCH_2_ and C—CH_2_), 3.16 (d, 1H, ^3^*J* = 12.0, N—CH_2_), 5.00 (s, 1H, OH). Anal. Calcd for C_15_H_25_NO_2_: C, 71.67; H, 10.02; N, 5.57. Found: C, 71.83; H, 9.77; N, 5.41.

### Refinement

The hydrogen atom of the hydroxy group was localized in the difference Fourier map and refined isotropically with fixed isotropic displacement parameters *U*_iso_(H) = 1.5*U*_eq_(O). The other hydrogen atoms were placed in calculated positions with C—H = 0.98–1.00Å and refined in the riding model with fixed isotropic displacement parameters *U*_iso_(H) = 1.5*U*_eq_(C) for the methyl group and 1.2*U*_eq_(C) for the other groups.

### Crystal data

**Table:**

C_15_H_25_NO_2_	*F*(000) = 552
*M**_r_* = 251.36	*D*_x_ = 1.264 Mg m^−^^3^
Monoclinic, *P*2_1_/*n*	Melting point = 437–439 K
Hall symbol: -P 2yn	Mo *K*α radiation, λ = 0.71073 Å
*a* = 5.8438 (2) Å	Cell parameters from 6340 reflections
*b* = 18.5756 (7) Å	θ = 2.2–32.6°
*c* = 12.2148 (5) Å	µ = 0.08 mm^−^^1^
β = 95.116 (1)°	*T* = 100 K
*V* = 1320.66 (9) Å^3^	Prism, colourless
*Z* = 4	0.30 × 0.30 × 0.20 mm

### Data collection

**Table:**

Bruker APEXII CCD diffractometer	3852 independent reflections
Radiation source: fine–focus sealed tube	3221 reflections with *I* > 2σ(*I*)
Graphite monochromator	*R*_int_ = 0.028
φ and ω scans	θ_max_ = 30.0°, θ_min_ = 2.0°
Absorption correction: multi-scan (*SADABS*; Sheldrick, 2003)	*h* = −8→8
*T*_min_ = 0.976, *T*_max_ = 0.984	*k* = −26→26
17142 measured reflections	*l* = −17→17

### Refinement

**Table:**

Refinement on *F*^2^	Primary atom site location: structure-invariant direct methods
Least-squares matrix: full	Secondary atom site location: difference Fourier map
*R*[*F*^2^ > 2σ(*F*^2^)] = 0.037	Hydrogen site location: calc
*wR*(*F*^2^) = 0.102	H atoms treated by a mixture of independent and constrained refinement
*S* = 1.00	*w* = 1/[σ^2^(*F*_o_^2^) + (0.055*P*)^2^ + 0.4*P*] where *P* = (*F*_o_^2^ + 2*F*_c_^2^)/3
3852 reflections	(Δ/σ)_max_ < 0.001
167 parameters	Δρ_max_ = 0.48 e Å^−^^3^
0 restraints	Δρ_min_ = −0.18 e Å^−^^3^

### Special details

**Table:**

Geometry. All s.u.'s (except the s.u. in the dihedral angle between two l.s. planes) are estimated using the full covariance matrix. The cell s.u.'s are taken into account individually in the estimation of s.u.'s in distances, angles and torsion angles; correlations between s.u.'s in cell parameters are only used when they are defined by crystal symmetry. An approximate (isotropic) treatment of cell s.u.'s is used for estimating s.u.'s involving l.s. planes.
Refinement. Refinement of *F*^2^ against ALL reflections. The weighted *R*–factor *wR* and goodness of fit *S* are based on *F*^2^, conventional *R*–factors *R* are based on *F*, with *F* set to zero for negative *F*^2^. The threshold expression of *F*^2^ > σ(*F*^2^) is used only for calculating *R*–factors(gt) *etc*. and is not relevant to the choice of reflections for refinement. *R*–factors based on *F*^2^ are statistically about twice as large as those based on *F*, and *R*–factors based on ALL data will be even larger.

### Fractional atomic coordinates and isotropic or equivalent isotropic displacement parameters (Å^2^)

**Table:**

	*x*	*y*	*z*	*U*_iso_*/*U*_eq_	
O1	0.72882 (11)	0.10966 (4)	0.39294 (6)	0.01342 (14)	
H1	0.847 (2)	0.1345 (7)	0.3939 (11)	0.020*	
C1	0.47868 (14)	0.21494 (5)	0.37839 (7)	0.00990 (16)	
C2	0.38858 (15)	0.19624 (5)	0.49006 (7)	0.01161 (17)	
H2A	0.5143	0.1741	0.5384	0.014*	
H2B	0.3421	0.2412	0.5257	0.014*	
N3	0.19197 (13)	0.14659 (4)	0.47886 (6)	0.01124 (15)	
C4	0.26546 (15)	0.07765 (5)	0.43377 (7)	0.01177 (17)	
H4A	0.1356	0.0432	0.4293	0.014*	
H4B	0.3919	0.0570	0.4834	0.014*	
C4A	0.34662 (15)	0.08849 (5)	0.31979 (7)	0.01051 (16)	
H4C	0.2157	0.1089	0.2712	0.013*	
C5	0.41334 (16)	0.01620 (5)	0.27117 (7)	0.01355 (18)	
H5A	0.5389	−0.0059	0.3197	0.016*	
H5B	0.2797	−0.0168	0.2678	0.016*	
C6	0.49189 (17)	0.02548 (5)	0.15586 (8)	0.01609 (18)	
H6A	0.3619	0.0430	0.1053	0.019*	
H6B	0.5416	−0.0216	0.1283	0.019*	
C7	0.69080 (17)	0.07913 (5)	0.15765 (8)	0.01640 (19)	
H7A	0.7326	0.0867	0.0817	0.020*	
H7B	0.8264	0.0588	0.2014	0.020*	
C8	0.62793 (16)	0.15179 (5)	0.20703 (7)	0.01312 (17)	
H8A	0.5039	0.1747	0.1586	0.016*	
H8B	0.7634	0.1840	0.2108	0.016*	
C8A	0.54791 (15)	0.14266 (5)	0.32298 (7)	0.01031 (16)	
C9	0.11013 (17)	0.13529 (5)	0.58766 (7)	0.01506 (18)	
H9A	0.0565	0.1811	0.6158	0.023*	
H9B	0.2360	0.1166	0.6381	0.023*	
H9C	−0.0167	0.1006	0.5818	0.023*	
O1'	0.18262 (12)	0.23557 (4)	0.22802 (6)	0.01587 (15)	
C1'	0.29057 (15)	0.25807 (5)	0.31084 (7)	0.01110 (17)	
C3'	0.69183 (15)	0.26497 (5)	0.40043 (8)	0.01269 (17)	
H3A	0.8080	0.2399	0.4507	0.015*	
H3B	0.7602	0.2732	0.3302	0.015*	
C4'	0.63912 (16)	0.33810 (5)	0.45054 (8)	0.01341 (18)	
H4D	0.5814	0.3308	0.5235	0.016*	
H4E	0.7818	0.3670	0.4609	0.016*	
C5'	0.45977 (16)	0.37870 (5)	0.37603 (8)	0.01372 (18)	
H5C	0.5237	0.3905	0.3058	0.016*	
H5D	0.4210	0.4244	0.4117	0.016*	
C6'	0.24123 (16)	0.33311 (5)	0.35295 (8)	0.01422 (18)	
H6C	0.1635	0.3288	0.4214	0.017*	
H6D	0.1351	0.3581	0.2978	0.017*	

### Atomic displacement parameters (Å^2^)

**Table:**

	*U*^11^	*U*^22^	*U*^33^	*U*^12^	*U*^13^	*U*^23^
O1	0.0096 (3)	0.0127 (3)	0.0174 (3)	0.0011 (2)	−0.0015 (2)	0.0010 (2)
C1	0.0095 (4)	0.0095 (4)	0.0106 (4)	0.0001 (3)	0.0002 (3)	−0.0001 (3)
C2	0.0127 (4)	0.0115 (4)	0.0106 (4)	−0.0004 (3)	0.0008 (3)	−0.0006 (3)
N3	0.0123 (3)	0.0107 (3)	0.0110 (3)	−0.0001 (3)	0.0026 (3)	0.0001 (3)
C4	0.0127 (4)	0.0104 (4)	0.0124 (4)	0.0005 (3)	0.0019 (3)	0.0009 (3)
C4A	0.0109 (4)	0.0095 (4)	0.0111 (4)	−0.0003 (3)	0.0007 (3)	−0.0004 (3)
C5	0.0169 (4)	0.0101 (4)	0.0138 (4)	−0.0005 (3)	0.0022 (3)	−0.0010 (3)
C6	0.0218 (5)	0.0125 (4)	0.0144 (4)	−0.0008 (3)	0.0042 (3)	−0.0031 (3)
C7	0.0189 (4)	0.0149 (4)	0.0164 (4)	−0.0001 (3)	0.0071 (3)	−0.0024 (3)
C8	0.0148 (4)	0.0120 (4)	0.0129 (4)	−0.0012 (3)	0.0036 (3)	−0.0005 (3)
C8A	0.0101 (4)	0.0099 (4)	0.0108 (4)	0.0006 (3)	0.0003 (3)	0.0004 (3)
C9	0.0172 (4)	0.0168 (4)	0.0117 (4)	0.0010 (3)	0.0040 (3)	0.0019 (3)
O1'	0.0163 (3)	0.0156 (3)	0.0149 (3)	0.0008 (2)	−0.0029 (2)	−0.0001 (2)
C1'	0.0104 (4)	0.0107 (4)	0.0124 (4)	−0.0004 (3)	0.0022 (3)	0.0019 (3)
C3'	0.0107 (4)	0.0111 (4)	0.0161 (4)	−0.0007 (3)	0.0001 (3)	−0.0015 (3)
C4'	0.0134 (4)	0.0114 (4)	0.0153 (4)	−0.0015 (3)	0.0005 (3)	−0.0016 (3)
C5'	0.0157 (4)	0.0106 (4)	0.0149 (4)	0.0000 (3)	0.0019 (3)	−0.0004 (3)
C6'	0.0128 (4)	0.0120 (4)	0.0176 (4)	0.0023 (3)	0.0000 (3)	−0.0012 (3)

### Geometric parameters (Å, º)

**Table:**

O1—C8A	1.4362 (10)	C7—C8	1.5363 (13)
O1—H1	0.829 (14)	C7—H7A	0.9900
C1—C1'	1.5393 (12)	C7—H7B	0.9900
C1—C2	1.5449 (12)	C8—C8A	1.5400 (12)
C1—C3'	1.5581 (12)	C8—H8A	0.9900
C1—C8A	1.5726 (12)	C8—H8B	0.9900
C2—N3	1.4701 (11)	C9—H9A	0.9800
C2—H2A	0.9900	C9—H9B	0.9800
C2—H2B	0.9900	C9—H9C	0.9800
N3—C9	1.4671 (11)	O1'—C1'	1.2175 (11)
N3—C4	1.4730 (11)	C1'—C6'	1.5223 (12)
C4—C4A	1.5237 (12)	C3'—C4'	1.5326 (12)
C4—H4A	0.9900	C3'—H3A	0.9900
C4—H4B	0.9900	C3'—H3B	0.9900
C4A—C5	1.5328 (12)	C4'—C5'	1.5250 (13)
C4A—C8A	1.5458 (12)	C4'—H4D	0.9900
C4A—H4C	1.0000	C4'—H4E	0.9900
C5—C6	1.5296 (13)	C5'—C6'	1.5373 (13)
C5—H5A	0.9900	C5'—H5C	0.9900
C5—H5B	0.9900	C5'—H5D	0.9900
C6—C7	1.5297 (13)	C6'—H6C	0.9900
C6—H6A	0.9900	C6'—H6D	0.9900
C6—H6B	0.9900		
			
C8A—O1—H1	109.8 (9)	C7—C8—C8A	111.54 (7)
C1'—C1—C2	107.88 (7)	C7—C8—H8A	109.3
C1'—C1—C3'	107.69 (7)	C8A—C8—H8A	109.3
C2—C1—C3'	108.16 (7)	C7—C8—H8B	109.3
C1'—C1—C8A	114.36 (7)	C8A—C8—H8B	109.3
C2—C1—C8A	108.00 (7)	H8A—C8—H8B	108.0
C3'—C1—C8A	110.56 (7)	O1—C8A—C8	109.05 (7)
N3—C2—C1	112.58 (7)	O1—C8A—C4A	104.79 (7)
N3—C2—H2A	109.1	C8—C8A—C4A	109.95 (7)
C1—C2—H2A	109.1	O1—C8A—C1	108.20 (7)
N3—C2—H2B	109.1	C8—C8A—C1	114.37 (7)
C1—C2—H2B	109.1	C4A—C8A—C1	110.01 (7)
H2A—C2—H2B	107.8	N3—C9—H9A	109.5
C9—N3—C2	108.70 (7)	N3—C9—H9B	109.5
C9—N3—C4	110.10 (7)	H9A—C9—H9B	109.5
C2—N3—C4	109.19 (7)	N3—C9—H9C	109.5
N3—C4—C4A	110.58 (7)	H9A—C9—H9C	109.5
N3—C4—H4A	109.5	H9B—C9—H9C	109.5
C4A—C4—H4A	109.5	O1'—C1'—C6'	119.70 (8)
N3—C4—H4B	109.5	O1'—C1'—C1	123.99 (8)
C4A—C4—H4B	109.5	C6'—C1'—C1	116.31 (7)
H4A—C4—H4B	108.1	C4'—C3'—C1	114.31 (7)
C4—C4A—C5	110.42 (7)	C4'—C3'—H3A	108.7
C4—C4A—C8A	111.17 (7)	C1—C3'—H3A	108.7
C5—C4A—C8A	111.15 (7)	C4'—C3'—H3B	108.7
C4—C4A—H4C	108.0	C1—C3'—H3B	108.7
C5—C4A—H4C	108.0	H3A—C3'—H3B	107.6
C8A—C4A—H4C	108.0	C5'—C4'—C3'	110.61 (7)
C6—C5—C4A	111.44 (7)	C5'—C4'—H4D	109.5
C6—C5—H5A	109.3	C3'—C4'—H4D	109.5
C4A—C5—H5A	109.3	C5'—C4'—H4E	109.5
C6—C5—H5B	109.3	C3'—C4'—H4E	109.5
C4A—C5—H5B	109.3	H4D—C4'—H4E	108.1
H5A—C5—H5B	108.0	C4'—C5'—C6'	110.64 (7)
C5—C6—C7	110.51 (8)	C4'—C5'—H5C	109.5
C5—C6—H6A	109.5	C6'—C5'—H5C	109.5
C7—C6—H6A	109.5	C4'—C5'—H5D	109.5
C5—C6—H6B	109.5	C6'—C5'—H5D	109.5
C7—C6—H6B	109.5	H5C—C5'—H5D	108.1
H6A—C6—H6B	108.1	C1'—C6'—C5'	112.77 (7)
C6—C7—C8	111.71 (8)	C1'—C6'—H6C	109.0
C6—C7—H7A	109.3	C5'—C6'—H6C	109.0
C8—C7—H7A	109.3	C1'—C6'—H6D	109.0
C6—C7—H7B	109.3	C5'—C6'—H6D	109.0
C8—C7—H7B	109.3	H6C—C6'—H6D	107.8
H7A—C7—H7B	107.9		
			
C1'—C1—C2—N3	66.10 (9)	C1'—C1—C8A—O1	178.11 (7)
C3'—C1—C2—N3	−177.68 (7)	C2—C1—C8A—O1	−61.79 (8)
C8A—C1—C2—N3	−58.00 (9)	C3'—C1—C8A—O1	56.37 (9)
C1—C2—N3—C9	−176.76 (7)	C1'—C1—C8A—C8	56.36 (10)
C1—C2—N3—C4	63.10 (9)	C2—C1—C8A—C8	176.46 (7)
C9—N3—C4—C4A	178.93 (7)	C3'—C1—C8A—C8	−65.38 (9)
C2—N3—C4—C4A	−61.80 (9)	C1'—C1—C8A—C4A	−67.95 (9)
N3—C4—C4A—C5	−177.62 (7)	C2—C1—C8A—C4A	52.15 (9)
N3—C4—C4A—C8A	58.53 (9)	C3'—C1—C8A—C4A	170.31 (7)
C4—C4A—C5—C6	179.09 (7)	C2—C1—C1'—O1'	−111.77 (9)
C8A—C4A—C5—C6	−57.05 (10)	C3'—C1—C1'—O1'	131.70 (9)
C4A—C5—C6—C7	56.19 (10)	C8A—C1—C1'—O1'	8.39 (12)
C5—C6—C7—C8	−55.41 (11)	C2—C1—C1'—C6'	68.10 (9)
C6—C7—C8—C8A	55.69 (10)	C3'—C1—C1'—C6'	−48.43 (10)
C7—C8—C8A—O1	59.18 (9)	C8A—C1—C1'—C6'	−171.74 (7)
C7—C8—C8A—C4A	−55.19 (9)	C1'—C1—C3'—C4'	52.23 (10)
C7—C8—C8A—C1	−179.54 (7)	C2—C1—C3'—C4'	−64.11 (9)
C4—C4A—C8A—O1	62.26 (9)	C8A—C1—C3'—C4'	177.83 (7)
C5—C4A—C8A—O1	−61.17 (9)	C1—C3'—C4'—C5'	−58.21 (10)
C4—C4A—C8A—C8	179.33 (7)	C3'—C4'—C5'—C6'	55.80 (10)
C5—C4A—C8A—C8	55.90 (9)	O1'—C1'—C6'—C5'	−129.56 (9)
C4—C4A—C8A—C1	−53.84 (9)	C1—C1'—C6'—C5'	50.56 (10)
C5—C4A—C8A—C1	−177.27 (7)	C4'—C5'—C6'—C1'	−52.24 (10)

### Hydrogen-bond geometry (Å, º)

**Table:**

*D*—H···*A*	*D*—H	H···*A*	*D*···*A*	*D*—H···*A*
O1—H1···N3^i^	0.830 (12)	2.195 (12)	2.8967 (10)	142.3 (12)

Symmetry code: (i) *x*+1, *y*, *z*.
